# Cold Autoimmune Hemolytic Anemia due to High-grade non Hodgkin's B cell Lymphoma with Weak Response to Rituximab and Chemotherapy Regimens

**Published:** 2015-07-01

**Authors:** Behzad Nazel Khosroshahi, Mohammad Jafari, Hossein Vazini, Alireza Ahmadi, Keivan Shams, Mahdi Kholoujini

**Affiliations:** 1 Emam Reza Hospital, Hamadan University of Medical Sciences, Hamadan, Iran; 2 Blood transfusion research center, High institute for research and Education in Transfusion Medicine, Tehran, Iran; 3 Department of Pathology, School of Medicine, Hamadan University of Medical Sciences, Hamadan, Iran; 4 Department of nursing, Hamedan branch, Islamic Azad University, Hamedan, Iran; 5 Department of Hematology and Oncology, School of Medicine, Hamadan University of Medical Sciences, Hamadan, Iran; 6 Department of Immunology, School of Medicine, Shiraz University of Medical Sciences, Shiraz, Iran; 7 Department of Mycology, School of Medicine, Tarbiat Modares University, Tehran, Iran

**Keywords:** Cold autoimmune hemolytic anemia, High-grade, non-Hodgkin's lymphoma, Rituximab, Chemotherapy

## Abstract

Autoimmune hemolytic anemia (AIHA) is characterized by shortening of red blood cell (RBC) survival and the presence of autoantibodies directed against autologous RBCs. Approximately 20% of autoimmune hemolytic anemia cases are associated with cold-reactive antibody. About half of patients with AIHA have no underlying associated disease; these cases are termed primary or idiopathic. Secondary cases are associated with underlying diseases or with certain drugs. We report herein a rare case of cold autoimmiune hemolytic anemia due to high-grade non-Hodgkin's lymphoma of B-cell type with weak response to rituximab and chemotherapy regimens. For treatment B cell lymphoma, Due to lack of treatment response, we used chemotherapy regimens including R- CHOP for the first time, and then Hyper CVAD, R- ICE and ESHAP were administered, respectively.

For treatment of autoimmune hemolytic anemia, we have used the corticosteroid, rituximab, plasmapheresis and blood transfusion and splenectomy. In spite of all attempts, the patient died of anemia and aggressive lymphoma nine months after diagnosis. To our knowledge, this is a rare report from cold autoimmune hemolytic anemia in combination with high-grade non-Hodgkin's lymphoma of B-cell type that is refractory to conventional therapies.

## Introduction

 Immune hemolytic anemias are disorders in which erythrocytes survival are reduced because of the deposition of immunoglobulin or complement on the red cell membrane. The immune hemolytic anemias can be grouped according to the presence of autoantibodies, alloantibodies or drug related antibodies. The autoimmune hemolytic anemias (AIHAs) are due to an altered immune response, resulting in the production of antibody against the host’s own erythrocytes, with subsequent hemolysis. The incidence of AIHA is estimated at 10–30 cases per 1 million populations.^1^ Approximately 20% of cases of AIHA are cold autoimmune hemolytic anemia.^2^ The hemolysis is predominantly intravascular and mediated by IgM auto-antibodys and complement proteins.^3^ In a study of 1834 patients, approximately 40% of cases of AIHA have been associated with an underlying disease, while the remainder were idiopathic.^4^ The causes of the production of autoantibody in patients with AIHA are various. Autoimmune antibodies, particularly cold-reacting antibodies, are sometimes produced following an infection or immune defects or lymphoprolifrative disorders.

The lymphomas are a heterogeneous group of malignancies that originate in a single lymphocyte that has undergone transforming mutations Conferring a growth and survival advantage in comparison to its normal cellular counterparts. The neoplasm usually originates in a lymph node, or lymphatic tissue in other sites (extranodal lymphoma), and can be localized or widespread at the time of diagnosis. The lymphomas are major causes of warm and cold autoimmune hemolytic anemia. Rituximab is a humanized monoclonal antibody directed against CD20. Binding of rituximab to cells expressing CD20 results in cell death via a combination of antibody-dependent cell cytotoxicity, complement activation and apoptosis. Rituximab results in depletion of both normal and malignant B cells.^5^^,^^6^ Moreover, rituximab is among the most important drugs for treatment of non-Hodgkin's lymphomas^7^ and warm autoimmune hemolytic anemias,^8^ particularly cold autoimmune hemolytic anemias.^9^

## CASE PRESENTATION:

**Figure 1:**
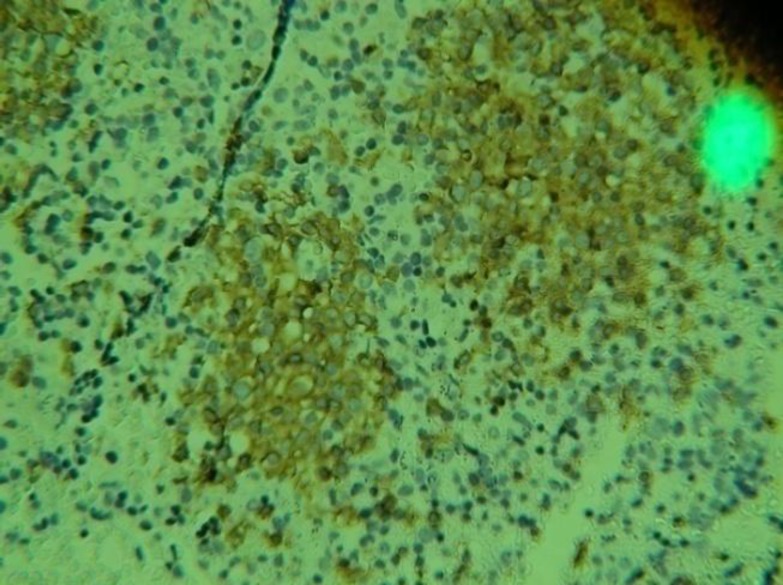
CD20 strongly positive in tumoral cells by immunohystochemistery panel

**Figure 2:**
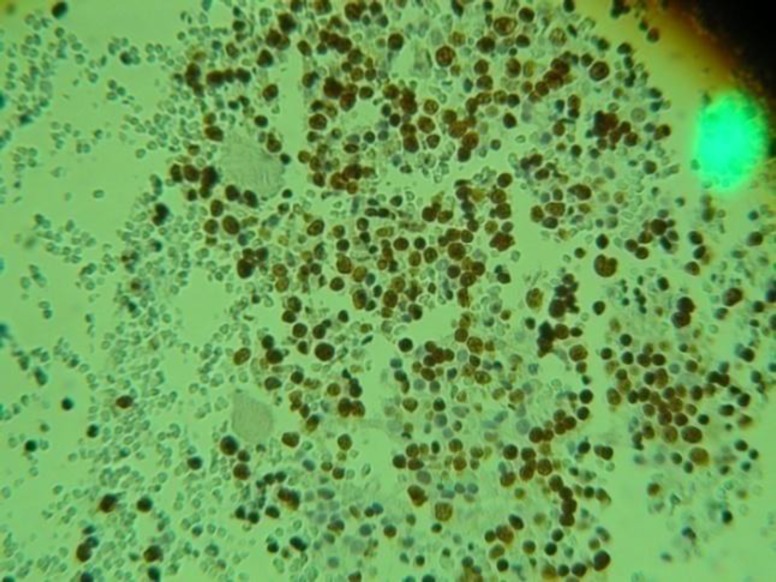
Ki67 strongly positive in more than 80 percent of tumoral cells by immunohystochemistery panel

 A 26-year-old Iranian white male referred to our hospital with severe anemia and generalized lymphadenopathy. To determine the cause of lymphadenopathy, a biopsy was taken from inguinal and neck lymph nodes. Pathological gross description of these specimens showed that both specimens had outer surface gray-white, solid and homogenous appearance. Microscopic description showed effacement of architecture with loss of follicles. The section showed monomorphous population of tumor cells with moderate small size, hyperchromatic round nuclei, scant cytoplasm, high N/C ratio, numerous mitosis and nodal capsules that were relatively intact. Immunohistochemistery studies showed that CD3 negative, CD20 positive, BCL-2 focal positive, cyclin-D1 negative, TDT few scattered positive in tumoral cells and Ki67 were strongly positive in more than 80 percent of tumoral cells (see Figures 1 and 2). Finally, immunehistochemistery (IHC) staining confirmed the diagnosis of high-grade non-Hodgkin's B-cell lymphoma (NHL). In microscopic observation from bone marrow aspiration and biopsy, cellurarity was normal and in deep sections small focal infiltration to moderate lymphocytic cells determined that could be due bone marrow involvement.The other hematopoetic cell lines were normal.

Clinical laboratory results of blood specimen are as follows: 

White Blood Cell (WBC): 11×10^9^/L, Red Blood Cell (RBC): 1.8×10^12^/L, Platelet (PLT): 177 ×10^9^/L, Haemoglobin (Hb): 5.7gr/dl, Haematocryte (HCT): 17.3 %, Mean Cell Volume (MCV): 96 fl, Mean Cell Haemoglobin (MCH): 32pg. Mean Cell Haemoglobin Concentration (MCHC): 33gr/dl. Reticulocyte count was 2.7%, Lactate Dehydrogenase (LDH): 4759 IU/L (normal range 200-400 IU/L), Total bilirubin: 5mg/dl (normal range0.5-1.5 mg/dl), Direct bilirubin: 1.8 mg/dl (normal range0.1-0.5 mg/dl), Direct Antiglobulin Test (DAT) was positive for complement C3. The peripheral blood smear showed RBC agglutination. 

To diagnose autoantibodies, screening antibody test was performed. The result was auto anti I. For treatment of lymphoma in this patient, chemotherapy regimens including R-CHOP (Rituximab, cyclophosphamide, vincristine, doxorubicin, prednisone), Hyper CVAD (cyclophosphamide, vincristine, doxorubicin, dexamethasone alternating with high dose methotrexate, cytarabine), R-ICE (Rituximab, ifosfamide, carboplatin, etoposide) and ESHAP (etoposide, methylprednisolone, cisplatin, cytarabine) were used, respectively. Each chemotherapy regimen was used for 3 courses with intervals of 21 days. For treatment of Anemia, prednisolone as corticosteroid (1mg/kg for 5days with chemotherapy and 15 daily continuously after chemotherapy) was used, the initial dose should be given 1-2 mg/kg body weight daily. The dose may be given once daily if tolerated and should be continued for 10-14 days according to response. Rituximab (375 mg/m^2^ by vein every 3 weeks), Plasmapheresis (40cc/kg/day for 3courses that each course was10 day) and Blood transfusion were used. Finally, due to undesirable response to above mentioned therapies, splenectomy was performed. In spite of all attempts patient died of anemia and aggressive lymphoma after nine months of diagnosis (one month after splenectomy). 

## Discussion

 In AIHA associated with the production of cold-type autoantibody, erythrocytes are usually coated with IgM.^2^ Under these circumstances, the fixation of complement frequently occurs^3^ Cold agglutinin disease represents approximately 20% of AIHA.^2^ In some cases, cold agglutinin disease is associated with a lymphoproliferative disorder, immune defect or infection. The cold antibody is usually an IgM with anti-I or less frequently anti-i specificity.^10^ The occurrence of autoimmune hemolytic anemia in patients with non-Hodgkin's lymphoma is well known. Cold autoimmiune hemolytic anemia is a complication in 1.1-4.8% of all non-Hodgkin's lymphoma.^6^ Sallah et al. have reported that autoantibody production in patients with non-Hodgkin's lymphoma is caused by several abnormalities including: 1) failure of immune tolerance mechanisms to eliminate immature lymphocytes that have been exposed to certain antigenic determinants on the surface of red blood cells and platelets 2) autoreactive clone activation due to genetic abnormalities (BCL-2, c-myc and others) , systemic viral infections or because of yet unknown events 3) defects in the apoptotic pathways such as FAS/Fas L and others may lead to an excessive accumulation of a T-cell clone. A neoplastic T cell may promote antibody production by B cell through negative signaling or excessive cytokine release.^11^ Numerous studies on the use of rituximab, a humanized monoclonal antibody, against CD20 showed cell death in both normal and malignant B cells and high efficacy of rituximab for treatment of non-Hodgkin's lymphomas and autoimmiune hemolytic anemias, particularly cold autoimmiune hemolytic anemias.^5^^,^^7^^,^^8^^,^^9^^,^^11^ Several reports have described the use of rituximab for treatment of primary and chronic cold autoimmiune hemolytic anemias.^9^

In a clinical study, Berensten et al. tested the use of rituximab in 86 patients with cold agglutinin disease. 66 out of 86 patients had lymphoma in this study. Of the 66 patients with lymphoma, 50 (76%) had non-Hodgkin's B-cell lymphoma; 60% of whom responded to rituximab as a single agent or in combination therapy.^14^ In a study conducted by D'Arena G et al.( 2007) on 11 patients treated with Rituximab, 8 achieved complete response (CR) and 3 partial response (PR). All patients remained in CR / PR at a median follow-up of 604 days.^12^ In a single-arm, open-label, prospective and multicentre clinical trial administration, rituximab was found to be effective for the treatment of chronic cold agglutinin and autoimmune haemolytic anaemia (CAD). The overall response rate was 45% (n=20), with one CR and 8 with PR. Patients with idiopathic CAD accounted for one CR and 3 PR (4 of 13; 31%), while patients with secondary CAD accounted for 5 PR (5 of 7; 71%). Median time to maximal response was 3 months (range: 1-5 months). Median duration of response was 6.5 months (range: 2-10 months) Six patients relapsed within 48 weeks of follow-up. ^13^

In spite of previous studies, our patient didn’t respond satisfactorily to treatment with rituxamab. For treatment of B cell lymphoma, we used conventional high-dose chemotherapy regimens including R- CHOP, Hyper-CVAD, R- ICE and ESHAP.

Adding rituximab to chemotherapy regimens was not found effective despite the reports presented on rituximab efficacy in treatment intermediate and high-grade non-Hodgkin's B-cell lymphoma.^7^ Regarding high resistance of patient to above-mentioned treatments, we used plasmapheresis to eliminate autoantibodies, blood transfusion with minimum in compatibility and finally splenectomty to increase heamoglobin of patient. Unfortunately, despite of all attempts, the patient died of aggressive lymphoma nine months after diagnosis. To our knowledge, this is a rare report from cold autoimmune hemolytic anemia in combination with high-grade non-Hodgkin's lymphoma of B-cell type that was refractory to varied treatments. 
